# Identification and functional analysis of two novel connexin 50 mutations associated with autosome dominant congenital cataracts

**DOI:** 10.1038/srep26551

**Published:** 2016-05-24

**Authors:** Yinhui Yu, Menghan Wu, Xinyi Chen, Yanan Zhu, Xiaohua Gong, Ke Yao

**Affiliations:** 1 Eye Center, Second Affiliated Hospital of Zhejiang University School of Medicine, Hangzhou, China; 2 Zhejiang Provincial Key Lab of Ophthalmology, Hangzhou, China; 3 School of Optometry and Vision Science Program, University of California, Berkeley, California, United States of America

## Abstract

Autosomal dominant congenital cataracts (ADCC) are clinically and genetically heterogeneous diseases. The present study recruited two Chinese families with bilateral nuclear cataract or zonular pulverulent phenotype. Direct sequencing of candidate genes identified two novel missense mutations of Cx50, Cx50P59A (c.175C > G) and Cx50R76H (c.227G > A), both co-segregated well with all affected individuals. Bioinformatics analysis predicted deleterious for both mutations. Functional and cellular behaviors of wild type and mutant Cx50 examined by stably transfecting recombinant systems revealed similar protein expression levels. Protein distribution pattern by fluorescence microscopy showed that Cx50R76H localized at appositional membranes forming gap junctions with enormous cytoplasmic protein accumulation, whereas the Cx50P59A mutation was found inefficient at forming detectable plaques. Cell growth test by MTT assay showed that induction of Cx50P59A decreased cell viability. Our study constitutes the first report that the Cx50P59A and Cx50R76H mutations are associated with ADCC and expands the mutation spectrum of Cx50 in association with congenital cataracts. The genetic, cellular, and functional data suggest that the altered intercellular communication governed by mutated Cx50 proteins may act as the molecular mechanism underlying ADCC, which further confirms the role of Cx50 in the maintenance of human lens transparency.

Congenital cataracts are defined as opacities of the lens that are present from birth, and are the leading cause of visual disability in children. About 8–25% of isolated congenital cataracts are hereditary^1^, most often in the autosomal dominant mode. Congenital cataracts exhibit high clinical and genetic heterogeneity. To date, over 39 genes and loci have been linked with the pathogenesis of congenital cataracts^2^,^3^. Of the disease-causing mutations reported, about half are located in crystallins and a quarter in gap junctions.

Gap junctions (GJs) play an important role in the formation of the extensive intercellular communication system for maintaining lens metabolic homeostasis, and hence transparency^4^,^5^. The GJs are transmembrane channels that provide vital pathways for the intercellular transport of ions and low-molecular-weight molecules with masses up to 1 kDa^6^,^7^. A GJ is formed by the docking of two connexons (hemichannels) from neighboring cells. A connexon, in turn, consists of six connexin (Cx) subunits, which can cluster at appositional membranes and form gap junction plaques between adjacent cells^8^.

Connexins are members of a multigene family, with at least 21 members that exhibit complex and overlapping patterns of expression^9^. In the human lens, Cx43, Cx46, and Cx50 (encoded as GJA1, GJA3, and GJA8, respectively) have been identified as critical for interconnecting lens fiber and epithelial cells^7^,^9^.

The importance of GJs for lens physiology is attested by the ocular abnormalities and cataractogenesis induced by mutations in both Cx46 and Cx50. The mechanisms proposed to account for the role of these mutations in the development of congenital cataracts include inefficiency in forming gap junction channels or impaired trafficking to the plasma membrane^10^,^11^,^12^,^13^,^14^,^15^,^16^,^17^,^18^,^19^, gain of hemichannel function^20^,^21^, alterations in voltage-dependent gating and permeability properties^22^, and dominant negative effects on wild type connexins^10^,^12^,^14^,^15^,^21^,^23^,^24^. A direct link between multiple mutations of Cx46 and Cx50 and congenital cataracts has been identified.

The present study was designed to characterize the cellular and functional properties of two novel Cx50 mutations that we identified in Chinese pedigrees associated with ADCC, and to gain further insights into the pathogenesis of inherited cataracts.

## Results

### Clinical findings

**Figure 1:**
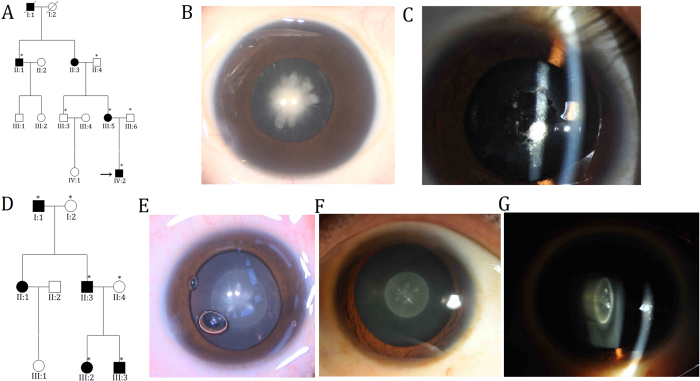
Pedigrees and phenotypes. (**A,D**) Pedigrees of families. Squares indicate men and circles women; black and white symbols represent affected and unaffected individuals, respectively. The proband is marked with an arrow, and asterisks indicate those members enrolled in this study. (**B,E**) Photographs of lens of the proband (IV:2 in Family 1 and III:3 in Family 2) presented as a dense congenital nuclear cataract and lamellar cataract involving the embryonal nucleus, respectively. Both were taken during cataract extraction. (**C**) Slit-lamp photograph of the right eye of the affected individual III:5 in Family 1, showing lens material absorption and pupillary membrane organization. (**F,G**) Front and oblique view of the same lens of the affected individual II:3 in Family 2, showing a perinuclear cataract with fine punctate opacities involving the central zone of the lens.

In Family 1, six members (three affected and three unaffected) participated in the study (Fig. 1A). The proband (IV:2) was a three-year-old boy with bilateral congenital nuclear cataracts, which are characterized as a central, dense nuclear opacity involving the embryonic and fetal nucleus of the lens (Fig. 1B). The proband’s mother (III:5) had suffered from bilateral lens opacities shortly after birth, and had undergone cataract extraction in the left eye at around seven years of age. A slit-lamp photograph of the untreated right eye revealed lens material absorption and pupillary membrane organization (Fig. 1C); thus, the cataract phenotype could not be defined definitely. Affected individual II:1 had undergone bilateral cataract surgeries; therefore, clinical findings showed no lens opacities. There was evidence of nystagmus and amblyopia in all affected individuals, suggesting severe visual deprivation in the critical period of eye development. Family 2 comprised three generations, with four affected members and two unaffected participants (Fig. 1D). The proband (III:3) and his father (II:3) exhibited almost the same appearance of lens opacity, which was described as bilateral zonular/lamellar with fine punctate located predominantly in the central zone (2 mm) of the lens (Fig. 1E,F). According to the medical records, affected individuals I:1 and III:2 had been diagnosed with bilateral congenital cataracts at birth and had cataract extraction performed. In both families, autosomal dominant inheritance was identified, and no participants showed any other ocular or systemic abnormalities.

### Mutation detection

**Figure 2:**
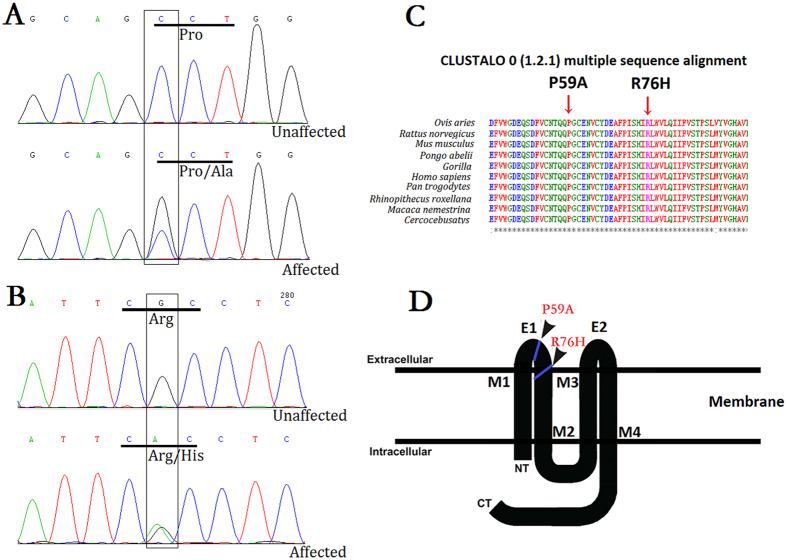
Mutation analysis in Cx50. (**A**) DNA sequence chromatograms of the affected members and unaffected members in Family 1 show a C/G transition at codon 59 that changed Pro(CCT) to Ala(GCT) (c.175C > G, p.P59A). (**B**) Sanger sequence of Cx50 showing a heterozygous G > A alteration, resulting in the substitution of an Arg(CGC) by His(CAC) at amino acid residue 76 (c.227G > A, p.R76H). (**C**) Multiple-sequence alignment of a section of the Cx50 amino acid sequence from different species revealed high conservation of Pro59 and Arg76 (indicated by red arrows). (**D**) Schematic diagram of the predicted domain structure of human Cx50 showing the positions at which the P59A and R76H mutations occur. As predicted, Cx50P59A lies within the first extracellular domain and Cx50R76H is located in the boundary of the first extracellular loop and the second transmembrane domain (indicated by solid black square). NT, NH2-terminus; CT, COOH-terminus; M1–M4, transmembrane domains; E1 and E2, extracellular loop domains.

Direct sequencing of the coding regions of candidate genes in affected individuals detected two novel heterozygous mutations in Cx50. In Family 1, a c.175C > G substitution was revealed, which led to the replacement of a proline at position 59 by alanine (p.P59A; Fig. 2A). The variant in Family 2 was identified as a c.227G > A transition that resulted in a missense mutation, where an arginine was replaced by a histidine at codon 76 (p.R76H; Fig. 2B). In both families, there was no noticeable disease-linked changes or any known nucleotide polymorphisms in the other candidate genes and both mutations showed a complete co-segregation with all affected individuals, neither mutation was not found in any of the unaffected family members or in the 100 unrelated normal controls.

### Potential function prediction

**Figure 3:**
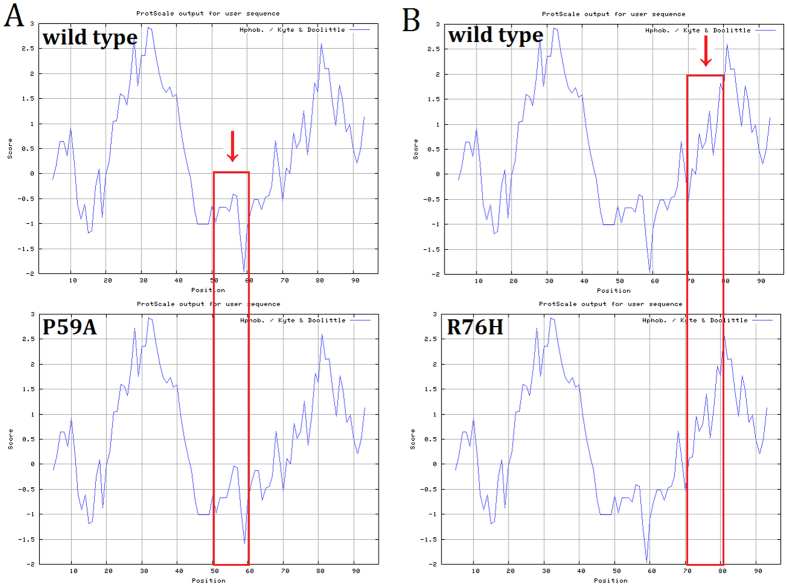
Hydrophilicity analysis of the wild type and mutant proteins. Protscale results shows slight change of hydrophilicity.

Multiple-sequence alignment indicated that Pro59 and Arg76, where the mutations occurred, were located within a phylogenetically conserved region of connexin 50 (Fig. 2C). PolyPhen-2 produced position-specific independent counts (PSIC) scores of 0.999 and 0.995, which are consistent with “probably damaging,” for P59A and R76H mutations, respectively; while the SIFT method revealed a score of 0.00 for both variants, indicating that the substitutions were predicted to affect protein function. PROVEAN analysis gave scores of −7.941 and −4.772 for P59A and R76H, respectively, which were predicted with high confidence to be “deleterious” for the protein. Mutation Taster predicted, with a probability value of 0.999 for each, that the pathogenic alterations may change the protein features of Cx50 and affect the splice site, further raising the likelihood of Cx50 dysfunction. Further predictions by PON-P2 and MutPred also were conducted. All the results are listed (Table 1). Moreover, in comparison with the wild type Cx50 protein, the hydrophobicity of the protein was changed slightly as a result of the mutations (Fig. 3). As the isolated predictive value of these programs can be increased by their combination, the data indicated that the mutations were likely deleterious and possibly contributed to the disease.

### Expression and subcellular localization of wild type and mutant Cx50 proteins in stably transfected cells

**Figure 4:**
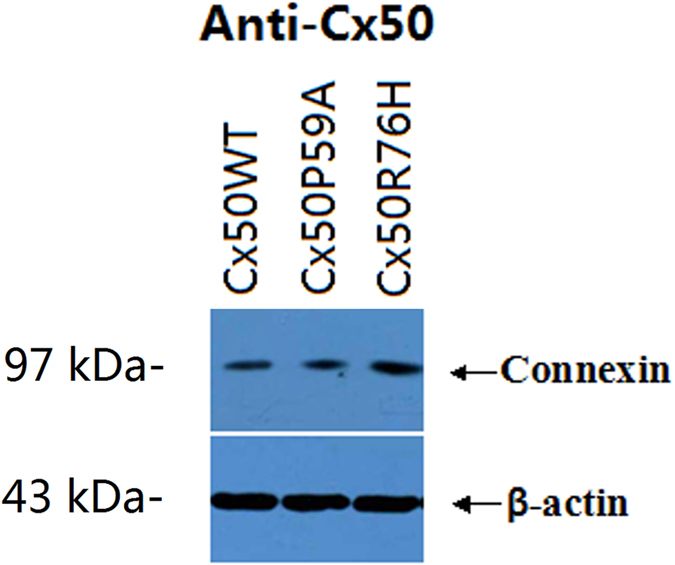
Western blot of the wild type and mutant Cx50 in stably transfected cells. Similar levels of protein with a band of 97 kDa were detected. The blots were probed with anti-Cx50 antibody. β-actin was used as a loading control.

**Figure 5:**
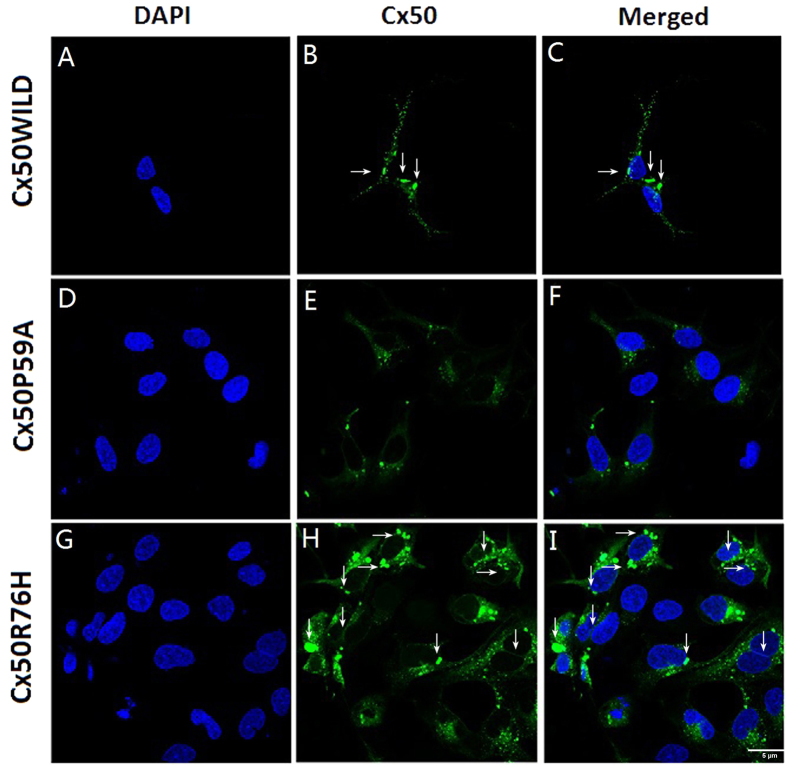
Sub-cellular localization pattern of Cx50 proteins. Both Cx50WT (**A–C**) and Cx50R76H (**G–I**) formed abundant gap junction plaques, as observed at appositional membranes and perinuclear cytoplasmic locations (arrows). Note the enormous aggregation blobs of Cx50R76H in the cytoplasm and plasma membrane (**H,I**). Images of Cx50P59A transfected cells showed a lack of gap junction plaques; instead, a very low level of expression protein was accumulated in the cytoplasm. Green: GFP; blue: DAPI staining of cell nuclei. The scale bar represents 5 μm.

GFP-tagged fusion constructs were stably expressed in Hek293 cells. Western blot analysis clearly showed similar levels of either wild type or mutant proteins in a band of approximately Mr ~97 kDa (Fig. 4). Fluorescent images showed that both Cx50WT (Fig. 5A–C) and Cx50R76H (Fig. 5G–I) recombinant proteins were localized at the cytoplasm perinuclear regions and appositional membranes, where they formed gap junction plaques. Typical punctate signals of intercellular GJs were observed. However, distinct from wild type Cx50, part of the Cx50R76H protein showed increased accumulation in cytoplasmic inclusions (described as enormous numbers of intracellular blobs), in addition to linear distribution along appositional membranes (Fig. 5H,I). Conversely, the Cx50P59A subunits alone failed to localize at sites of cell-cell apposition, a phenomenon consistent with the lack of gap-junction formation (Fig. 5D–F). Only a low level of Cx50P59A protein was detected within the cytoplasm.

### Cell viability evaluation

**Figure 6:**
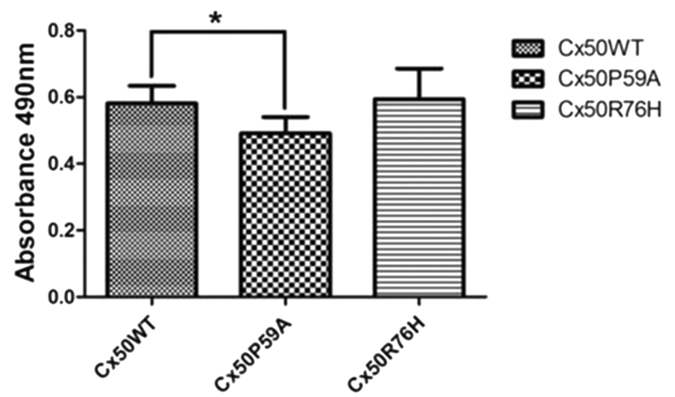
Quantitative analysis by MTT assay of cell viability. Induction of CxP59A expression decreased the proliferation and growth capability of cells. *P < 0.05.

The results of the MTT assays showed that there were no significant differences between the effects of Cx50WT and Cx50R76H on cell growth after 48 h (Cx50WT: 0.581 ± 0.053; Cx50R76H: 0.594 ± 0.091, *P* = 0.347), whereas a decrease in cell numbers was observed in Cx50P59A-transfected cells when compared with induction of the Cx50WT protein (Cx50WT: 0.581 ± 0.053; Cx50P59A: 0.491 ± 0.050, *P* = 0.000) (Fig. 6). Quantitative analysis revealed that the P59A mutation reduced cell growth and viability.

## Discussion

Our study is the first to have associated Cx50P59A and Cx50R76H mutations with ADCC. Three similar variants at the same codon of Cx46 (P59L^25^, R76H^26^, and R76G^27^) were reported previously to co-segregate with nuclear punctate, total, and lamellar pulverulent cataract phenotypes, respectively. The two Cx50 substitutions of P59A and R76H identified in the present study were associated with central nuclear and zonular punctate congenital cataracts, respectively, and were consistent with the phenotypes reported previously. The prevalence of changes in codons 59 and 76 indicates that the residues are mutation hotspots. According to similarities in the protein sequences of Cx46 and Cx50, P59 and R76 residues are well-conserved across species by multiple sequence alignment, which highlights the functional importance of this region. Both mutations co-segregated well with affected members, and were not detected in unaffected individuals or in the 100 normal controls (data not shown). Additionally, the mutations were predicted to be deleterious, with consistent results, by bioinformatics analysis; this strongly suggests that the substitutions can be disease causative.

Like other connexins, Cx50 is a membrane protein that contains four transmembrane domains (M1, M2, M3, M4) linked by two extracellular loops (E1, E2), which are the most conserved domains among connexins^23^,^28^,^29^, as well as a cytoplasmic loop and intracytoplasmic NH2 and COOH terminals (Fig. 2D). Connexins form connexon hemichannels in the cell membranes. These hemichannels dock in adjacent cells to form gap junctions, allowing the transport of molecules^30^. The transmembrane domains are believed to participate in the oligomerization into connexon hemichannels, which are essential for the correct transport of protein into a plasma membrane^31^. The NH2-terminus may interfere with the pore lining of the hemichannel, as well as mediating channel permeability and voltage gating^32^. Furthermore, the extracellular loops are believed to play a key role in both the docking of intercellular connexin hemichannels, and thus the formation of gap junction channels, and the voltage gating that regulates the channel^33^,^34^.

Given that the P59A and R76H substitutions were located within the phylogenetically conserved motif of the E1 loop and the boundary of E1 and the M2 domain, respectively (Fig. 2D), which are the sites that provide strong interaction between adjacent cells, and so enable intercellular transport of molecules^12^, we speculated that the mutations may affect normal intercellular connexon docking, thereby resulting in incorrect transport of proteins. To dissect the precise pathogenic mechanisms of the two Cx50 mutations, further studies were conducted by stably transfecting recombinant DNA constructs into Hek293 cells to characterize the cellular and functional consequences of the mutations.

Western blot analysis revealed similar expression levels for the wild type and mutant Cx50 proteins, with a band of the expected size of 97 kDa (Fig. 4), indicating that the mutations did not result in instability of the protein. We then examined the subcellular localization of the Cx50 proteins. Earlier studies have shown that wild type Cx50 is localized at the plasma membrane and appositional membranes^10^,^15^. Confocal microscopy detected both wild type and Cx50R76H mutant proteins, primarily in the cytoplasmic perinuclear region and in the appositional membranes, where a significant number of gap junction plaques formed. This suggests that trafficking of the protein was not affected by the Cx50R76H mutation. The amino acid sequence at the interface between the transmembrane domain and the extracellular loop is thought to play an important role in inter-hemichannel interactions, channel gating, and regulation^10^. All connexins contained a positively-charged amino acid (either arginine [R] or lysine [K]) at the corresponding position, where the R76H mutation occurred. The substitution of Arg by His in this boundary (with both being positively-charged amino acids) might not alter the charge on the surface of the extra cellular loop; if not, the connexon docking would not be affected, which may help to explain the fact that this mutation did not affect the transport of the proteins nor their capacity to form gap junction plaques. Our finding was consistent with the trafficking fates of four previously described, cataract-associated connexin 50 mutations involving the E1 and M1 loop junction (R23K^10^, V44E^12^, W45S^21^, and G46V^20^,^21^), which imposed no disruptive effect on oligomerization and, consequently, trafficking, with the result that no cellular or functional consequence was found.

However, in addition to the linear distribution along appositional membranes, an increased intensity of Cx50R76H mutant blobs was observed accumulating in the cytoplasmic inclusions, which may suggest a different localization pattern with wild type Cx50 protein. This distribution pattern was in agreement with another Cx50 mutation (p.H277Y, P88T)^19^,^35^, in which increased accumulation of mutant protein was found in the cytoplasm. We presume that the aberrant protein accumulations may lead to altered function of gap channels and metabolic imbalance of the lens, which might be a contributing factor to cataract formation, although further functional studies are still required to elucidate the precise pathogenic mechanisms.

In contrast, Cx50P59A failed to form gap junction plaques at appositional membranes, instead expressing at a very low level throughout the cytoplasmic inclusions. This meant that the mutation inhibited the ability of connexins to form gap junction channels, thereby impairing intercellular communication. Studies have shown that most mutations in amino acids within the Cx50 E1 loop result in loss of intercellular coupling, and act as a loss-of-function mutation (E48K, D47H, D47A, and D47N)^12^,^13^,^23^; our finding further validated the important role of the E1 loop in the docking of connexins responsible for the formation of functional gap junction channels. In the same way, we believe that the replacement altered the docking capability of Cx50 hemichannels to form gap junction channels, thereby leading to impaired trafficking of Cx50 to the plasma membrane. And the consequent decrease in lens intercellular communication may result in disarrangement or separation of lens fiber cells, which, in turn, may cause metabolic disorder of the lens and ultimately may lead to cataract formation.

There are reports that connexin may control cell growth, and so may be implicated in cell proliferation^36^,^37^; therefore, we investigated the effects of the mutations on cell viability via MTT assay. Our quantitative analysis revealed that stable ectopic expression of mutated CxR76H did not affect cell growth, whereas induction of Cx50P59A introduced a negative effect on cell growth when compared with the Cx50 wild type control (Fig. 6). We presume that the loss-of-function activity, along with the decrease in cell growth and proliferation, may cause a metabolic imbalance within the lens that ultimately triggers the development of cataracts. At the cellular level, we cannot rule out the effect of a complex sequence of events, including hemichannel function, loss of membrane potential, and disruption of ion gradients, on cell viability. Thus, further experiments on functional defects may elucidate the precise mechanisms of cataract formation.

The avascular lens has developed an extensive intercellular communication system, via gap junction channels, to facilitate direct transfer of molecules between adjacent cells for the preservation of tissue homeostasis and the maintenance of lens transparency. The central role of connexins in lens transparency has been confirmed by the identification of a number of Cx46 and Cx50 mutations associated with congenital cataracts. To date, at least 34 heterozygous mutations of Cx50 have been reported to induce genetic cataracts (summarized in Table 2). The majority of these mutations occur in the extracellular loop and the first/second transmembrane domains, which are associated with gap-junction formation and pore structure, respectively. The detected E1/E2 mutations probably share a similar mechanism that compromises the binding of connexins. Ten mutations (G46R, G46V, D47A, D47H, D47N, D47Y, E48K, S50P, V64G/A, and S73F) in the E1 loop have been shown to be associated with congenital cataracts, which highlights the functional importance of this region and the surrounding residues. The molecular consequences that have been characterized functionally are summarized (Table 2). Most of these exhibit impaired trafficking, thus leading to alteration of intercellular communication, and several loss-of-function mutations may alter gating and, possibly, permeability of channels. Other effects may include activation of intracellular stress responses and formation of aberrant hemichannels, or a dominant negative effect. The diverse mutational mechanisms further illuminate the role of Cx50 in lens transparency.

Hence, we consider the Cx50P59A and Cx50R76H variations to be causative mutations of ADCC in the pedigrees. However, the ultimate effect of mutations on gap junction channels cannot be explained by our model. Whether the two mutations may be a dominant negative inhibitor of wild type Cx50 or impact voltage-dependent gating in hemichannels remains to be investigated. Because there are many different derangements in the cellular behaviors of the Cx50 mutants, further functional study is required to elucidate the pathophysiological consequences of the mutations.

In conclusion, we identified two novel missense mutations within conserved P59 and R76 of Cx50 that are associated with ADCC. Functional analysis showed that the mutation of Cx50P59A is inefficient at forming gap junction plaques, does not induce intercellular communication, and leads to decreased cell viability and proliferation. Conversely, Cx50R76H subunits were targeted properly to plasma membrane regions of cell-cell interaction while increasing the cytoplasmic accumulation, a possible underlying mechanism for cataract formation. Together, these changes may disrupt the intercellular communication mediated by gap junction channels and lead subsequently to the development of congenital cataracts. Our study expanded the mutation spectrum of Cx50 that is associated with congenital cataracts, provided further insight into the connexin channel function and understanding of the roles of intercellular communication in the lens, and also confirmed that Cx50 is important in the maintenance of lens transparency. Further experiments on this cataract-related genetic defect will improve our understanding of the mechanism of cataract formation.

## Materials and Methods

### Clinical evaluation and DNA specimens

This study was approved by the medical ethics committees of the Second Affiliated Hospital to Zhejiang University, Hangzhou, China, and was conducted in accordance with the tenets of the Declaration of Helsinki. Two families of Chinese origin with ADCC were recruited. All affected and unaffected individuals underwent detailed ophthalmic examinations. The phenotypes were documented using slit-lamp photography after pupillary dilatation. Peripheral blood samples were collected after informed written consent had been obtained from the participants. Genomic DNA was extracted from lymphocyte leukocytes using a QIAamp DNA Blood Mini Kit (Qiagen, Hilden, Germany), according to the manufacturer’s instructions. A total of 100 unrelated, ethnically-matched subjects without congenital cataracts were also recruited as controls.

### Mutation screening and sequencing

A functional candidate approach was performed for the screening of disease-causing mutations, using previously described primers and polymerase chain reaction (PCR) conditions^13^. The PCR products were bi-directionally sequenced using an ABI PRISM^TM^ Big Dye^®^ Terminator v3.1 Cycle Sequencing Kit (Applied Biosystems, Foster City, CA, USA) and analyzed using Chromas 1.62, which compared the products to reference sequences in the National Center for Biotechnology Information (NCBI) GenBank database.

### Bioinformatics analysis

Multiple sequence alignment was performed using the Basic Local Alignment Search Tool for conservation analysis. Online biology tools, including PolyPhen-2 (Polymorphism Phenotyping), SIFT (Sorting Intolerant from Tolerant), PROVEAN (Protein Variation Effect Analyzer), and MutationTaster were used to predict the potential functional impact of each amino acid substitution on proteins. Moreover, the possible effects on protein function were analyzed using the PON-P2 (http://structure.bmc.lu.se/PON-P2/) and MutPred (http://mutpred.mutdb.org/) *in silico* prediction programs. In addition, hydrophobic properties of the wild type and mutant Cx50 were analyzed by the online Protscale tool (http://web.expasy.org/protscale/).

### Generation of constructs

Wild type human Cx50 cDNA was subcloned into the expression vector pEGFP-N1 as the template for further cloning steps. Quick-change site-directed mutagenesis was performed to construct the mutant plasmids in accordance with the manufacturer’s protocol, and the products were purified with a QIAfilter Plasmid Maxi Kit (Qiagen). The desired sequence of resultant constructs (Cx50WT, Cx50P59A, and Cx50R76H) was further confirmed by DNA sequencing, so as to verify fidelity.

### Cell culture and stable transfection

Human Embryonic Kidney 293 (Hek293) cells were maintained in Roswell Park Memorial Institute (RPMI)-1640 medium, and were supplemented with 10% fetal bovine serum (FBS) in a humidified atmosphere containing 5% CO_2_ at 37 °C. Transfection was carried out using Lipofectamine 2000 (Invitrogen) according to the manufacturer’s instructions. Stably-transfected cell clones expressing either wild type or mutant Cx50 were selected with respect to their resistance to 800 μg/ml Geneticin (G418) (Invitrogen). To maintain stability of the Cx50 expression, G418 were applied during cell culturing in RPMI-1640.

### Immunoblotting

Cells at about 90% confluence were collected in ice-cold phosphate buffered saline (PBS) and centrifuged at 1000 rpm at 4 °C for 5 min. Total protein was extracted from the stably transfected Hek293 cells by means of lysis buffer supplemented with protease inhibitors (Sangon Biotech, Shanghai, China). The protein concentration was estimated using a bicinchoninic acid (BCA) assay kit (Sangon Biotech, Shanghai, China). After incubation on ice for 30 min, the extracts were centrifuged at 14,000 rpm at 4 °C for 15 min, followed by resolving using 10% SDS-polyacrylamide gel electrophoresis and transferring to polyvinylidene difluoride membranes (Millipore, Billerica, MA). The extracts were subjected then to immunoblotting with a 1:1000 dilution anti-Cx50 rabbit polyclonal antibody (Santa Cruz Biotechnology, Dallas, TX, USA), as well as a 1:5000 dilution anti-β-actin antibody (Sigma-Aldrich, St. Louis, MO, USA) at 4 °C, overnight. Fluorescent secondary antibody (Cell Signaling Technology, Danvers, MA, USA) at 1:5000 dilution was used for incubation. The blots were analyzed using a ChemiDoc^TM^ MP imaging system (Bio-Rad Laboratories, Hercules, CA, USA), in which the expression levels of the target protein were normalized relative to β-actin expression.

### Fluorescence and confocol imaging

Stably transfected Hek293 cells that expressed the required green fluorescent protein (GFP)-tagged connexins were grown on glass coverslips (BD BioSciences, San Jose, CA) in a 12-well chamber for 24 hours. Cells at about 90% confluence were fixed with 4% paraformaldehyde at room temperature for 15 min, permeabilized with 0.1% Triton X-100 and 2% bovine serum albumin (BSA) in PBS, and then blocked in solution. DAPI (4′,6-diamidino-2-phenylindole) (Sigma-Aldrich) was used to label the cellular nuclei, followed by six washes of PBS. Cellular distributions of the proteins were analyzed using a laser confocal scanning microscope (Leica TCS SP8, Leica Microsystems, Wetzlar, Germany) using 63X/1.4 NA oil immersion objectives. Composite figures were assembled using Adobe Photoshop software (Adobe Systems, San Jose, CA).

### MTT assay

To test directly whether the mutation would affect cell viability, a MTT (3-[4,5-Dimethylthiazol-2-yl]-2,5-diphenyltetrazolium bromide) assay was performed to compare the effects of induced Cx50 expression on cell numbers. Stably transfected cells were seeded into a 96-well plate (10^4^ cells/well) for 48 hours, and then were subjected to the assay using a Cell Proliferation Kit I(MTT) (Roche, Indianapolis, IN, USA) according to the manufacturer’s instructions. The viability of the cells was assessed by measuring the absorbance at 492 nm using a Bio-Rad iMark Microplate Absorbance Reader (Bio-Rad Laboratories). Each experiment was repeated three times.

## Additional Information

**How to cite this article**: Yu, Y. *et al*. Identification and functional analysis of two novel connexin 50 mutations associated with autosome dominant congenital cataracts. *Sci. Rep.* **6**, 26551; doi: 10.1038/srep26551 (2016).

## Figures and Tables

**Table 1:** *In silico* predictions of functional effects for the two mutations identified in this study.

Prediction program		Cx50P59A	Cx50R76H
SIFT	Value (<0.05)	0.00	0.00
	Prediction	Not tolerated	Not tolerated
Polyphen-2	Score	0.999	0.995
	Prediction	probably damaging	probably damaging
PROVEAN	Score	−7.941	−4.772
	Prediction	deleterious	deleterious
Mutation Taster	Value	0.999	0.999
	Prediction	disease causing change the protein features	disease causing affected the splice site
PON-P2	Probability for pathogenicity	0.401	0.686
	Standard error	0.069	0.068
	Prediction	Unknown	Unknown
MutPred	Probability of deleterious mutation	0.810	0.952
	Molecular mechanisms disrupted	Gain of sheet (P = 0.1208) Loss of glycosylation at T56 (P = 0.2738) Gain of catalytic residue at P59 (P = 0.4651) Loss of disorder (P = 0.4751) Loss of stability (P = 0.5064)	Gain of glycosylation at S73 (P = 0.0849) Loss of stability (P = 0.1046) Gain of helix (P = 0.132) Loss of catalytic residue at R76 (P = 0.1676) Loss of sheet (P = 0.1907)

**Table 2:** Summary of mutations found in connexin50 associated with human congenital cataract.

AA change	Connexin domain	Cataract phenotype	Mechanisms	Reference
L7P	NT	–	has not been studied yet	38
R23T	NT	Nuclear	Impaired intracellular trafficking Dominant-negative inhibition of wt Cx50 Lack of GJ conductance and dye transfer activities	10
I31T	M1	Nuclear	Predict: proper trafficking	39,40
R32T	M1	Nuclear	has not been studied yet	41
T39R	M1	Bilateral cataract and microcornea	Predict: impaired trafficking	11,40
V44A	M1	Nuclear	Proper trafficking Impaired hemichannels gating	42
V44E	M1	Bilateral cataract and microcornea	Proper trafficking Dominant-negative inhibition of wt Cx50 Lack of GJ channel conductance fail to form functional GJ channels	12
W45S	M1	Jellyfish-like cataract and microcornea	Proper trafficking Dominant-negative inhibition of wt Cx50 Lack of GJ channel conductance	21
G46R	E1	Bilateral cataract and microcornea	Predict: impaired trafficking	11,40
G46V	E1	Total	Cytotoxicity due to enhanced hemichannel function proper trafficking	20,21
D47H	E1	Nuclear and zonular pulverulent	Predict: impaired trafficking	13,40
D47N	E1	Nuclear and pulverulent	Impaired intracellular trafficking Lack of GJ channel conductance Lack of hemichannel currents, GJ conductance, and dye transfer activities	12
D47Y	E1	Nuclear	Predict: impaired trafficking	13,40
E48K	E1	Zonular nuclear pulverulent	Impaired intercellular trafficking Dominant-negative inhibition of wt Cx50 Lack of GJ conductance Normal hemichannel function	23
V64G	E1	Nuclear	Predict: proper trafficking	40,43
S73F	E1	nuclear	has not been studied yet	44
V79L	M2	full moon like with Y-sutural opacities	Forming functional homotypic intercellular channels, Alteration in voltage gating and reduction in single-channel open probability Lower levels of conductance Changes in the properties of junctional coupling Proper trafficking Dominant-negative inhibition of wt Cx50	12
P88Q	M2	“Balloon-like”cataract with Y-sutural opacities or pulverulent	Impaired intercellular trafficking Lack of GJ channel conductance Dominant-negative inhibition of wt Cx50 Lack of hemichannel currents, GJ conductance, and dye transfer activities	14,45
P88S	M2	Zonular pulverulent	Impaired intercellular trafficking Dominant-negative inhibition of wt Cx50 Lack of GJ channel conductance and dye transfer activities Lack of hemichannel currents	15,22
P88T	M2	Total	Accumulation of mutant protein Increased cell growth	35
H98P	M2	–	has not been studied yet	38
P189L	E2	Nuclear cataract and microcornea	Predict: proper trafficking	40,46
V196M	E2	–	Predict: proper trafficking	40,47
R198Q	E2	Bilateral cataract and microcornea	has not been studied yet Predict: impaired trafficking	40,48
R198W	E2	Bilateral cataract and microcornea	has not been studied yet Predict: impaired trafficking	40,49
P199S	E2	–	Predict: proper trafficking	40,47
E201K	E2	Perinuclear	Impaired trafficking Decreased cell growth and viability Lack of hemichannel currents, GJ conductance, and dye transfer activities	16
ins670A	M4	Total	Impaired intercellular trafficking	18
ins776G	CT	Triangular	Impaired intercellular trafficking	17
S276F	CT	Pulverulent nuclear	Proper trafficking Inhibiting the function of GJ channel in a Dominant negative manner and the hemichannel function in a recessive negative manner	24
H277Y	CT	pulverulent nuclear	Impaired intercellular trafficking	19
L281C	CT	Zonular Cataract	has not been studied yet	50
